# Almanac 2015: atrial fibrillation research in Heart

**DOI:** 10.1136/heartjnl-2015-307809

**Published:** 2016-01-20

**Authors:** Muhammad Jawad-Ul-Qamar, Paulus Kirchhof

**Affiliations:** 1 Institute of Cardiovascular Sciences, University of Birmingham, Birmingham, UK; 2 SWBH NHS Trust, Birmingham, UK; 3 UHB NHS Trust, Birmingham, UK; 4 Atrial Fibrillation NETwork (AFNET), Münster, Germany; 5 Department of Cardiovascular Medicine, Hospital of the University of Münster, Münster, Germany

## Abstract

Atrial fibrillation continues to attract interest in the cardiovascular community and in *Heart*. Over 60 original research and review papers published in *Heart* in 2014–2015 cover various aspects of atrial fibrillation, from associated conditions and precipitating factors to new approaches to management. Here, we provide an overview of articles on atrial fibrillation published in *Heart* in 2014–2015, highlighting new developments, emerging concepts and novel approaches to treatment.

## Introduction

The 2014–2015 have been active years in atrial fibrillation (AF) research. Contributions from *Heart* on this subject have been very significant and substantial. The articles reflect the diverse nature of problems that patients with AF and their physicians are faced with, ranging from factors that allow to estimate the risk of cardiovascular complications and novel, hypothesis-generating associations of AF with biomarkers to insights into the optimal approach to anticoagulation, rate control and rhythm control therapy. We summarise some of the more interesting findings reported on AF in *Heart* in the year 2014 and 2015.

## Risk factors for developing AF and AF progression

### Baseline ECG, heart rate and age

A US-based registry (ORBIT -AF Outcomes Registry for Better Informed Treatment of Atrial Fibrillation.) analysed more than 6000 patients for risk of progression of AF from paroxysmal to persistent to permanent.^1^ It was found that increasing age by 10 (OR 1.16) and presence of AF in ECG at baseline (OR 2.30) were strong predictors of AF progression. Meanwhile, decreasing heart rate than 80 (OR 0.84) was protective against progression of AF.

### Ivabradine

**Figure 1:**
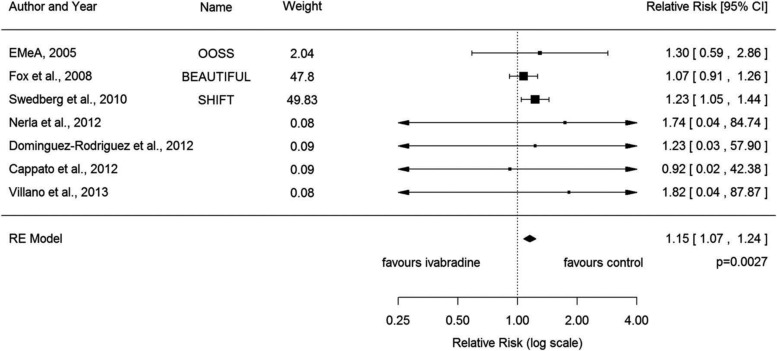
Forest Plot of RR of atrial fibrillation with ivabradine.^2^ RR, relative risk.

An important meta-analysis identified a small (relative risk (RR) 1.15) but relevant risk for developing AF in patients treated with ivabradine.^2^ This effect is incidentally observed in the same group of patients (baseline heart rate >70) that get the highest benefit from receiving ivabradine in terms of decreasing hospitalisation. This RR of developing AF could be attributed to change in the I_f_ current induced by ivabradine, a modification of the atrial resting membrane potential, or due to the potential proarrhythmic effects of bradycardia. Mechanistic studies are warranted to identify the mechanisms of AF induction by ivabradine (figure 1).

### Diastolic dysfunction

Results of subanalysis from Tromsø Study in Norway showed that severely enlarged atrial size as a marker for diastolic dysfunction was only echocardiographic marker associated with risk of developing AF (HR 4.2).^3^ This was independent of other mitral valve Doppler indices of diastolic dysfunction. Left atrial (LA) size increases with increasing diastolic dysfunction due to long-term change in left heart flow dynamics.

### Haemodialysis

**Figure 2:**
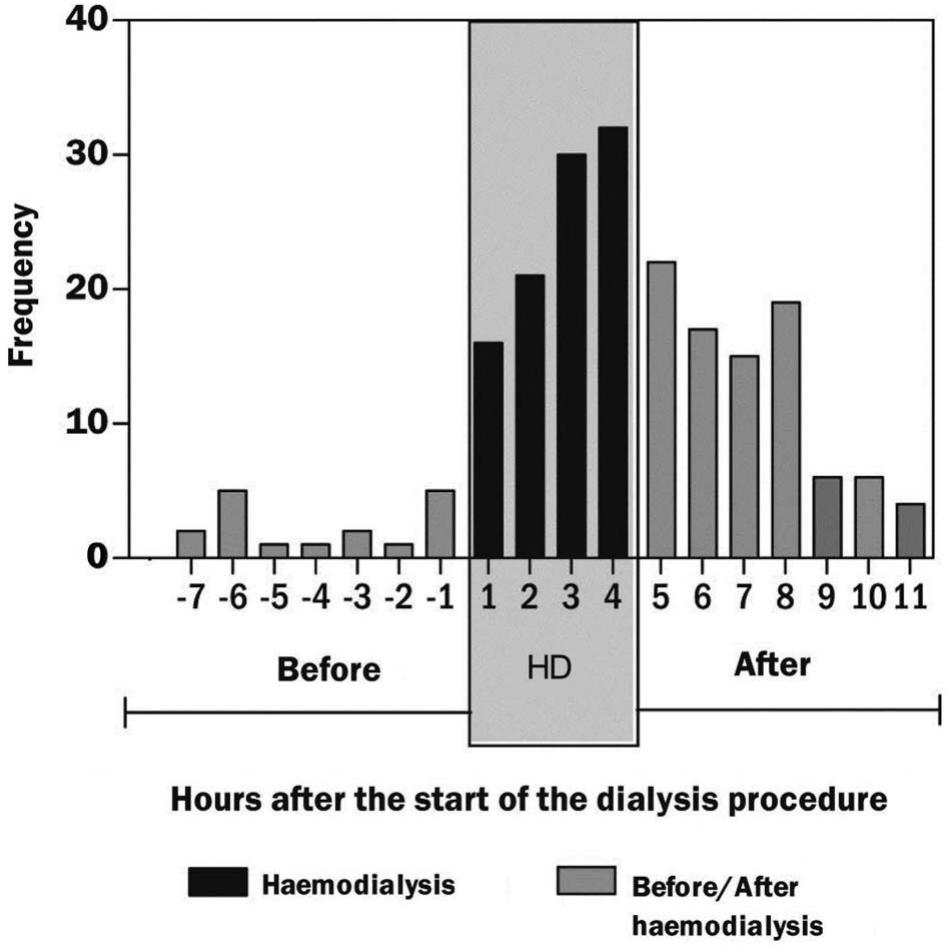
Onset of atrial fibrillation episodes on relation to start of haemodialysis.^4^

A hypothesis-generating analysis of a cohort of dialysis patients with an implanted pacemaker or defibrillator suggested that the haemodialysis procedure itself could trigger AF.^4^ The onset of atrial high-rate events (AHRE) clusters around the time of dialysis that can be considered as a proxy measure for AF. An association between higher exacted volume and lower dialysate potassium concentration is considered as triggers of AF. Comparatively, patients receiving peritoneal dialysis had less episodes of AF. These findings are consistent across other studies, which includes age, gender, coronary artery disease and atrial dimensions as additional risk for developing AF in haemodialysis patients (figure 2).^5^ ^6^

### Exercise

Several papers have assessed the impact of physical exercise on risk of developing AF.^7^ ^8^ There was an interesting Swedish study in more than 44 000 healthy adult men demonstrating a U-shaped relation between exercise and risk of AF.^9^ They found that moderate-to-severe exercise in leisure time or bicycling/walking for more than 60 min in a day was associated with a RR of 1.17 and 1.04 of developing AF later in life. Interestingly, this trend is seen to reverse in the older age with reduction in RR with similar levels of exercise. Similar favourable trends of exercise are seen for middle-aged and elderly women.^10^

It is well established that exercise capacity is reduced in AF with or without the presence of left ventricular systolic impairment.^11^ ^12^ There is a suggestion that paroxysmal atrial fibrillation (PAF) could add to the interplay between exercise and inherited conditions such as hypertrophic cardiomyopathy.^13^ Patients with PAF had a substantially lower exercise tolerance, even though they remained in Sinus Rhythm (SR) during exercise testing. After adjustment for age, sex and body mass index, PAF still had an independent RR of 4.65 for reduced exercise tolerance.

### No benefit of atrial septal defect closure

A Danish study for adult patients diagnosed with Atrial septal defect (ASD) shows that ASD closure gives rise to a higher risk of new AF (HR 8.4) as compared with age and gender matched comparison cohort, with a 10 year cumulative incidence of 11%.^14^ The risk of stroke was higher in patients with ASD with (HR 2) or without (HR 2.6) ASD closure, suggesting that AF is not prevented by ASD closure. This observation suggests that other factors than the altered haemodynamic function determine AF in patients with ASD. Alternatively, an even earlier timing of ASD closure may be needed to prevent AF.

### Other hypothesis-generating associations with AF

An analysis of Myocardial Ischemia National Audit Project database covering all PCI Percutaneous Coronary Intervention procedures in England and Wales for the short-term effects of air pollution on cardiovascular events in England and Wales was published in 2014.^15^ This showed an increased risk of hospital admissions due to AF and arrhythmias and high levels of NO_2_. A high content of particulate matter less than 2.5 µm in diameter was associated with increased mortality secondary to AF, arrhythmia and pulmonary embolism.

An observational study for risk of developing atrial flutter and fibrillation in patients admitted with pericarditis showed an incidence rate of 4.3% with more than 90% having an episode of AF in the first 24 h.^16^ All of them reverted to sinus rhythm. However, there was 35% 3 month recurrence rate for patients who initially developed AF. The authors advocated for anticoagulation in high-risk patients. No increased risk of pericardial tamponade with anticoagulant therapy was shown.

## Biomarkers in AF

Biomarkers have recently generated a lot of interest in prediction, diagnosis and prognostic risk stratification of AF.^17–20^

### Troponin I, natriuretic peptides and norepinephrine levels

**Figure 3:**
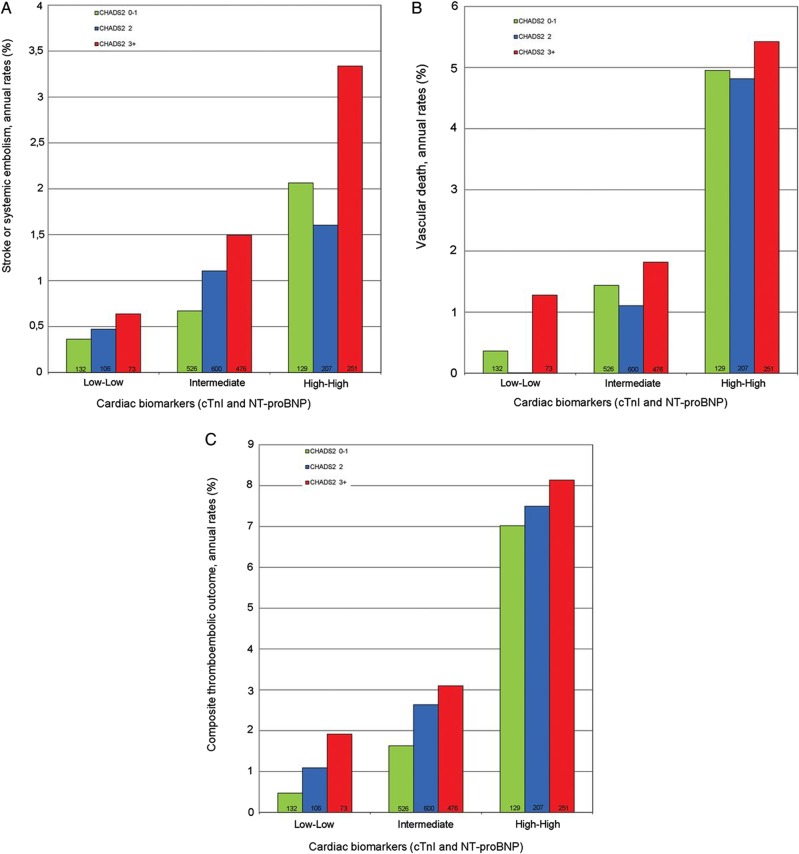
Study endpoints in relation to combined cardiac biomarker levels and CHADS_2_ score. Stroke or systemic embolism (A), vascular death (B) and composite thromboembolic outcome consisting of ischaemic stroke, systemic embolism, myocardial infarction, pulmonary embolism and vascular death (C).^21^ cTnI, cardiac troponin. NT-pro BNP, N Terminal-pro Brain Natriuretic Peptide. CHADS2, Stroke score (based on a 1 point each for Congestive heart failure, Hypertension, Age more than 75, Diabetes mellitus and 2 points for previous Stroke or TIA.)

An substudy of RE-LY (Randomized Evaluation of Longterm anticoagulant TherapY) regarding the prognostic value and risk stratification of biomarkers in AF has indicated an interesting use of cardiac biomarkers.^21^ They have indicated that serial high levels of cardiac troponin I (cTnI) and N Terminal-pro Brain Natriuretic Peptide (NT-proBNP) are associated with high incidence of stroke and systemic embolism (HR 4.54) and vascular death (HR 8.62). Others have earlier found similar association between these biomarkers and prognosis in AF.^22–25^ A Japanese study showed higher levels of atrial natriuretic peptide, brain natriuretic peptide and norepinephrine (NE) in persistent versus PAF.^26^ Only elevated NE levels were found to have an association with sick sinus syndrome (figure 3).

### Liver enzymes

Elevated circulating levels of liver enzymes are found to have moderately strong association with increased AF incidence in a large prospective community-based cohort study of more than 15 000 subjects.^27^ The association was linear and strongest for gamma glutamyl transferase (GGT) with doubling of GGT levels leading to 20% increase in AF risk after adjusting for the confounding factors. The association of AF incidence and aspartate amino transferase (AST) and to a lesser extent alanine amino transferase (ALT) showed a U-shaped curve with maximum incidence at the two extremes. This can be explained by right-sided heart failure leading to hepatic congestion or non-alcoholic fatty liver disease that increases the cardiovascular risk due to effect on glucose and lipid metabolism^28–30^ Correlation between deranged LFTs and risk of cardiovascular disease has also been published in a subanalysis from Framingham heart study.^31^

### Adiponectin

An association as a function of increasing age was seen between higher circulating adiponectin levels and risk of developing AF.^32^ This is paradoxical to the contemporary belief that higher adiponectin levels are cardioprotective. Further work is required in this field to identify a clear association with this novel biomarker.

## Epidemiology and risk factors

### Increasing incidence of AF in the UK

A large population-based cohort study looked at the discharge record of 2.2 million individuals aged 45 and above, from UK Clinical Practice Research Datalink.^33^ They found more than 91 000 incident AF cases. The incidence of AF has increased from 5.9/1000 person-years in 2001 to 6.9/1000 person-years in 2013. The overall incidence in terms of 1000 person-years increases with increasing age (25.1 for patients between 80–89) and is also higher for Caucasians (8.1) versus Asians (5.4) and African Americans (4.6).

### Predictive value of the CHADS_2_ and CHA_2_DS_2_Vasc scores for cardiovascular events in patients without AF

CHADS_2_ and CHA_2_DS_2_Vasc scores have been validated to predict the risk of stroke in patients with AF.^34^ ^35^ In a study, these scores were used to assess risk of new stroke/transient ischaemic attack (TIA) in the absence of AF in patients with an acute coronary syndrome (ACS).^36^ Both these scores showed a reasonable association predicted annual risk of stroke/TIA with an absolute annual incidence of 1% with CHADS_2_ ≥3 and CHA_2_DS_2_Vasc ≥4. This is in line with the results from another study by Poci *et al*^37^ that found association of CHADS_s_ with mortality and stroke. Others have shown association of these score with mortality after stroke, risk of developing new AF and risk of stroke or death after CABG.^38–40^

### AF and heart failure

AF and heart failure are often ‘vicious twins’, and each condition can worsen the other. A study in Tanzania showed that AF attributed to at least 16% cases of clinical heart failure presenting to tertiary-care hospital.^41^ This is an interesting finding if compared with the EPOCH (Epidemiology, Practice, Outcomes, and Costs of Heart Failure) study conducted in the USA in 2004 that compared epidemiological characteristics of hospitalised patients with heart failure and association of various comorbidities with different ethnicities, which showed AF was prevalent in 19.7% of African–Americans with heart failure as compared with 38.3% in Caucasian patients with heart failure.^42^ The report illustrates the global impact of AF and heart failure.

### AF as a predictor of increased mortality in low-gradient aortic stenosis

AF was found as independent predictor of mortality (HR 1.74) in patients with aortic stenosis (AS) in a large single centre observational study of severe AS treated with medical therapy, surgical aortic valve replacement or transcatheter aortic valve implantation.^43^ In general, the group receiving medical treatment had the worst prognosis with all-cause mortality of 81% at 3.9 years of follow-up. A study showed AF as a determinant of low-flow state in severe AS (OR 4.17).^44^ In another study, patients with severe low-gradient AS, AF was also associated with a poor prognosis and increased mortality.^45^ These observations highlight the importance to diagnose AF in patients with AS.

### Overall AF prevalence in hospitalised patients

An interesting cross-sectional survey in Belgium on a single day in a tertiary-care hospital identified total prevalence of AF at 16.8%.^46^ The presence of AF was associated with old age, hypertension and valvular heart disease. Interestingly, only 51% were appropriately treated with oral anticoagulation.

### Prognosis of silent AF after myocardial infarction

An observational study by Stamboul *et al* shows worse 1 year prognosis with increased hospital admissions and worsening heart failure for patients found to have silent AF within 2 days of admission with acute myocardial infarction (MI).^47^ It was previously suggested that silent AF was three times more common than symptomatic AF after acute MI.^48^ This highlights importance of continuous ECG monitoring for all patients with MI to detect AF.

## Screening for silent AF

Early diagnosis of AF is highly desirable to initiate therapy prior to the first complication.^49^ A review article sheds light on emerging techniques to screen for AF.^50^ Patient-operated ECG monitors or smartphone sensors may be suitable tools to screen for AF.^51–54^ In selected patients, long-term screening with implantable loop recorder can generate more AF detection.^55^ The need for AF-screening programmes, their optimal design and the best use of therapy (mainly oral anticoagulation) are not fully understood. Ongoing studies evaluating initiation of anticoagulation in patients with atrial high-rate episodes (eg, ARTESiA or NOAH—AFNET 6) and community screening programmes such as STROKESTOP^56^ ^57^ will provide further information in future.

## Imaging in AF

### Prognostic factors based on imaging parameters

**Figure 4:**
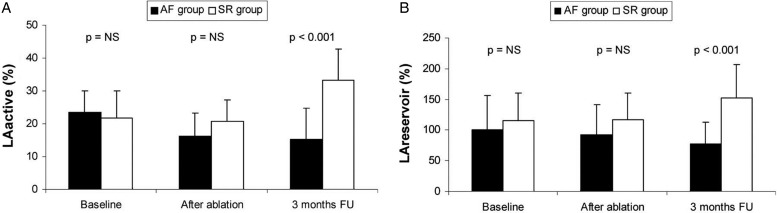
Changes in LA function after catheter ablation. SR group shows an improvement in LA reservoir function. AF, atrial fibrillation; LA, left atrial; NS, not significant.

A review article highlights several adverse prognostic features in imaging for AF.^58^ In two-dimensional conventional echocardiography, LA dilatation and left ventricular systolic function was shown to be associated with increased risk of stroke, heart failure and all-cause mortality. Mitral stenosis and hypertrophic cardiomyopathy (HCM) strongly also increases the risk of stroke in AF. In trans-oesophageal echocardiography (TOE), spontaneous echo contrast, LA thrombus and complex aortic valve (AV) plaque predict increased risk of systemic thromboembolism and stroke. In cardiac MRI (CMRI), favourable parameters were associated with decreased risk of stroke (OR 0.2). Moreover, LA conduit function as measured by CMRI was an important measure of success after catheter ablation (figure 4).

### Brain MRI to tailor anticoagulation therapy

**Figure 5:**
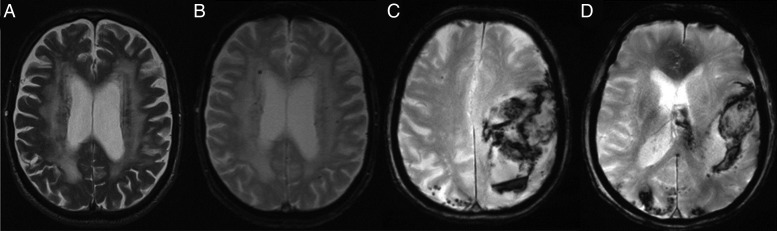
Patients with atrial fibrillation deemed unsuitable for oral anticoagulation T2 (A) and T2* (B) image at 1.5 T demonstrating confluent white matter hyperintensities and cerebral microbleeds in a patient with atrial fibrillation with recent transient ischaemic attack T2* images (C/D) at 3.0 T demonstrating an acute lobar haemorrhage and multiple cerebral microbleeds in a patient with atrial fibrillation with suspected cerebral amyloid angiopathy.^59^

Another review article looked into the value of brain MRI imaging to tailor anticoagulation therapy.^59^ It was found that silent brain infarction is commonly seen in patients with AF.^60^ ^61^ This is found to be dependent on the age of patient and on the type of AF. Presence of cerebral microbleeds (CMB), which are frequently found in patients with AF without prior stroke, should be taken as a risk for intracranial bleeding on anticoagulation. However, large studies are needed to confirm this observation. Number and location of CMB is affected by CHADS_2_ and CHA_2_DS_2_Vasc scores. This is mostly due to effect of vascular risk factors (figure 5).^62^

### Body Surface Potential Mapping and ECG imaging in AF

Use of ECG imaging, a novel fibrillation imaging modality using surface ECG combined with cardiac CT/MRI for the atrial anatomy to assess complexity of AF, was also highlighted.^63^ More recently, Body Surface Potential Mapping has been shown to identify the high-frequency sources in the atria without an imaging modality that were previously measured by invasive methods.^64^ This can lead to better patient selection before the actual ablation procedure based on the prediction of response to AF ablation therapy.

## Anticoagulation for stroke prevention

### Which antithrombotic therapy: NOACs, vitamin K antagonist, combination therapy

A large network meta-analysis of 20 studies combining more than 78 000 patients demonstrated that the new oral anticoagulants performed better in reducing the risk of stroke or venous embolism.^65^ Another meta-analysis with more than 100 000 patients showed 47% and 64% odds risk reduction of fatal bleeding with NOAC compared with vitamin K antagonist (VKA) therapy and low-molecular weight heparin (LMWH).^66^

It is well established that combined antiplatelet therapy and anticoagulation increases the risk of bleeding and that such ‘triple therapy’ should be confined to short periods of time in selected patients with ACS and/or recent PCI.^67^ ^68^ An analysis of large European data set identified a common mistake in antithrombotic therapy in patients with AF, that is, the continuation of aspirin therapy in patients with stable vascular disease.^69^ Far more than half of the patients subjected to combination therapy with aspirin and oral anticoagulation had no indication for combination therapy, putting them at unnecessary risk for bleeding.

The TIARA trial in 238 patients, comparing aspirin and anticoagulation in a cohort of patients with AF with moderate stroke risk and ‘favourable’ imaging characteristics on TOE, suggested that some patients may fare well without anticoagulation.^70^ They found that in the absence of LA thrombus on TOE, aspirin was non-inferior to VKA for all-cause mortality, stroke, ACS and major bleeding. Interestingly, TIA was not included as a primary end point, and seven patients suffered from TIA in the aspirin group. This highlights the challenges when considering individualised anticoagulation therapy in patients at low-to-moderate stroke risk. The results of this pilot study need validation by a larger scale trial.

### Anticoagulation in atrial flutter

A review article published in 2015, taking into account 52 published articles, indicated that atrial flutter renders a higher risk of thromboembolic complications.^71^ Imaging in atrial flutter also reveals high prevalence of spontaneous echo contrast and thrombus in left atrial appendage (LAA). Within the limitations of the small published data sets, this analysis underpins the current practice to offer anticoagulation to flutter patients.

## Ablation for AF

### Surgical ablation

A meta-analysis summarising the available information on surgical ablation for AF has been published.^72^ This extensive and first-of-its-kind meta-analysis suggests that surgical LA ablation is a safe and effective method of maintaining sinus rhythm for more than 1 year in patients with AF and undergoing concomitant cardiac surgery as compared with cardiac surgery alone.

### NOACs or warfarin in catheter ablation

A meta-analysis of 14 observational data sets compared dabigatran and warfarin for risks of thromboembolic events and major bleeds.^73^ There was no significant difference found between the two groups for either of the two end points. However, a numerical difference of more thromboembolic events in dabigatran group versus warfarin (0.55% dabigatran vs 0.17% warfarin, RR 1.78, 95% CI 0.66 to 4.80, p=0.26) was seen.

Another meta-analysis of observational studies also showed a non-significant but higher trend of worse neurological outcomes with dabigatran versus warfarin.^74^ No significant difference between dabigatran and warfarin was found in another meta-analysis.^75^ There is a need for controlled trials of uninterrupted anticoagulation regimes in patients with AF ablation.

### Change in renal function associated with arrhythmia recurrence

**Figure 6:**
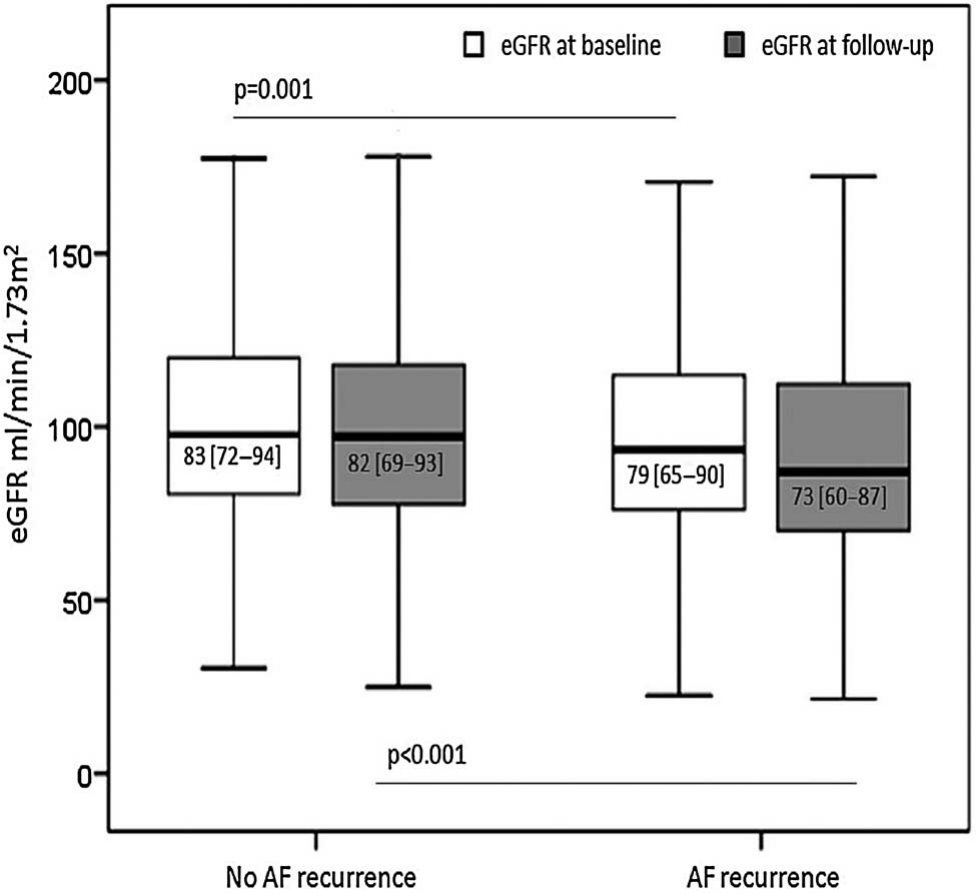
Estimated GFR in patients with and without recurrences at baseline and follow-up.^76^ AF, atrial fibrillation.

A German study with a cohort of 783 subjects found an associated with AF recurrence and worsening renal function (GFR).^76^ It was found that patients with recurrence had worse GFR on baseline and on follow-up. The higher CHADS_2_ and CHA_2_DS_2_Vasc scores were associated with worse renal functions. Finally, those patients who had decline in their GFR had more AF recurrences. This effect was independent of the type of OAC used. AF is known to decrease renal function.^77^ This may be possibly due to embolic phenomenon or haemodynamic mechanisms related to AF. Ablation and restoration of sinus rhythm generally improves renal function.^78^ However, deterioration of renal function may contribute to recurrent AF. Further studies are clearly needed to validate this novel observation (figure 6).

### Peri-procedural imaging in catheter ablation

**Figure 7:**
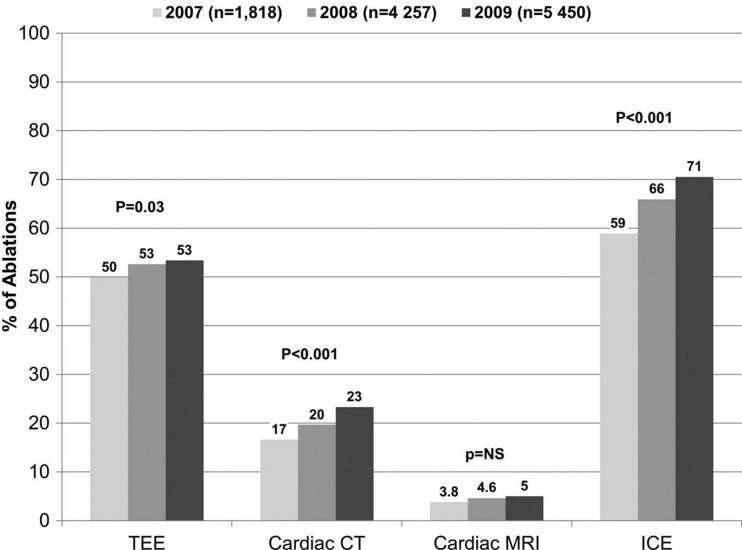
Temporal trends of imaging usage before (CT, MRI, TOE) and during (ICE) ablation.^79^ ICE, intracardiac echocardiography.

An observational study found an association between peri-procedural cardiac imaging such as TOE, intracardiac echocardiography (ICE), cardiac CT and MRI, and better outcomes in patients undergoing catheter ablation for AF.^79^ They found that use of preprocedural cardiac CT/MR was associated with a lower risk of TIA/stroke at 6 months (0.4% vs 0.9%, adjusted HR 0.46). Use of ICE was found to be associated with higher risk of bleeding (1.1% vs 0.7%, adjusted HR 1.76), but also with a lower incidence of repeat ablation (5.7% vs 8.5%, adjusted HR 0.68). Interestingly, TOE was not found to affect procedural outcomes. The usefulness of cardiac CT and MRI in identifying LA thrombus in patients undergoing ablation has been found in other studies^80^ ^81^ on top of their benefit in assessing cardiac anatomy and pulmonary vein size and location^82–84^ and chances of successful outcome (figure 7).^85^

## AF-related inappropriate shocks in patients with ICD

**Figure 8:**
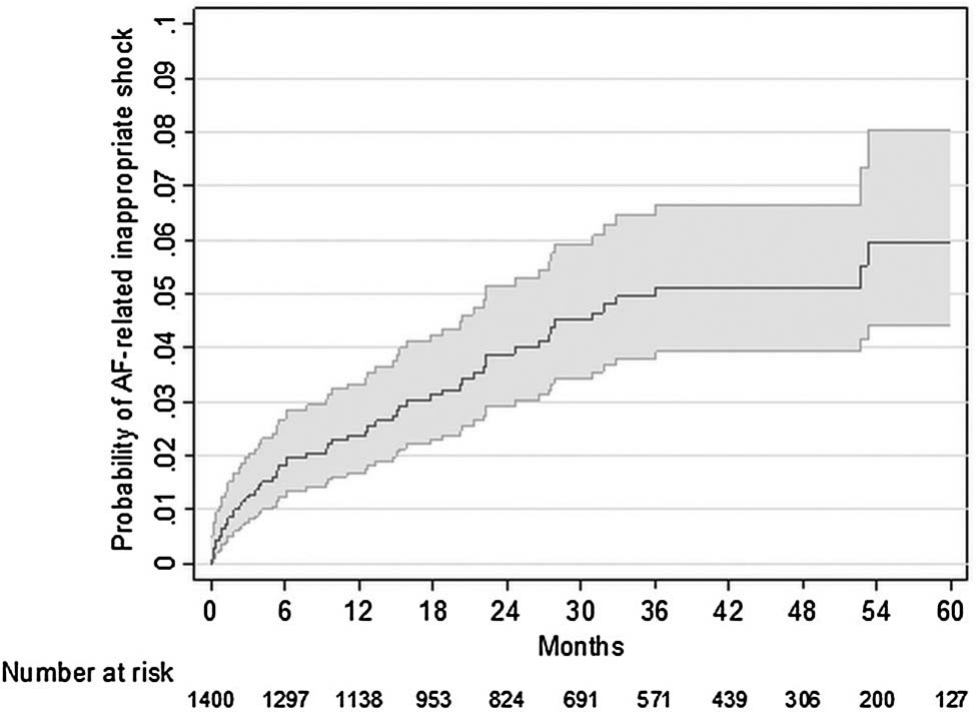
Kaplan–Meier estimation of probability of atrial fibrillation-related (AF-related) inappropriate shocks.^89^

It has been known that prolonged ECG monitoring with pacemakers and CRT-D have high detection rates of atrial high-rate episodes,^86^ ^87^ especially those with home monitoring that decreases the time to detect AHRE.^88^ Most, but not all of these episodes reflect paroxysmal, often undiagnosed (silent) AF. In a study of 1404 patients, it was found that AF lasting for more than 10 min is detected in a quarter of the patients who received CRT-D.^89^ Roughly, three-quarters of all inappropriate arrhythmia detections were due to AF, with 60 patients (4% of the total) receiving inappropriate shocks due to AF (2.69 patients/100 patient-years). Reprogramming of the shock criteria could have avoided many of these inappropriate shocks. Another French case report also explained the use of home monitoring to prevent in appropriate AF-related shocks (figure 8).^90^

In summary, AF remains one of the major topics of research published in *Heart*. We learned a lot, but there is a lot more to find out and study to improve outcomes in patients with AF in future.
